# MicroRNA Regulation in Renal Pathophysiology

**DOI:** 10.3390/ijms140713078

**Published:** 2013-06-25

**Authors:** Jianghui Hou, Dan Zhao

**Affiliations:** 1 Renal Division and Center for Investigation of Membrane Excitability Diseases, Washington University in St. Louis, 660 South Euclid Avenue, St. Louis, MO 63110, USA; 2 Division of Pharmacology, PLA 85th Hospital, 1328 Hua Shan Road, Shanghai 20052, China; E-Mail: zdandzjw@sina.com

**Keywords:** microRNA, kidney, diabetic nephropathy, hypercalciuria, hypertension

## Abstract

MicroRNAs are small, noncoding RNA molecules that regulate a considerable amount of human genes on the post-transcriptional level, and participate in many key biological processes. MicroRNA deregulation has been found associated with major kidney diseases. Here, we summarize current knowledge on the role of microRNAs in renal glomerular and tubular pathologies, with emphasis on the mesangial cell and podocyte dysfunction in diabetic nephropathy, the proximal tubular cell survival in acute kidney injury, the transport function of the thick ascending limb in Ca^++^ imbalance diseases, and the regulation of salt, K^+^ and blood pressure in the distal tubules. Identification of microRNAs and their target genes provides novel therapeutic candidates for treating these diseases. Manipulation of microRNA function with its sense or antisense oligonucleotide enables coordinated regulation of the entire downstream gene network, which has effectively ameliorated several renal disease phenotypes. The therapeutic potentials of microRNA based treatments, though promising, are confounded by major safety issues related to its target specificity, which remain to be fully elucidated.

## 1. Introduction

MicroRNAs comprise a large family of 21–22-nucleotide-long RNAs that have emerged as key post-transcriptional regulators of gene expression in animals and plants [1,2]. In animals, microRNAs are predicted to control the activity of ~50% of all protein-coding genes [3]. Functional studies indicate that microRNAs participate in the regulation of almost every cellular process, and are intrinsically associated with many human pathologies.

**Figure 1:**
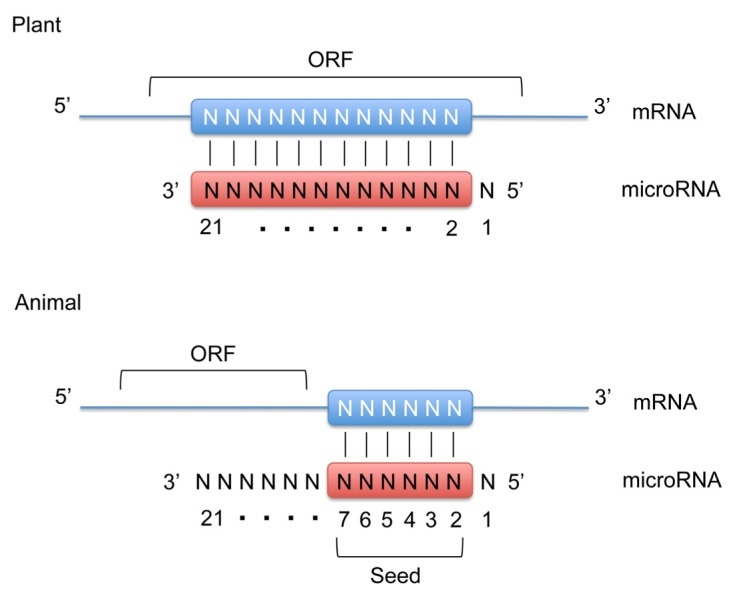
MicroRNA target recognition mechanism. Plant microRNAs recognize fully complementary binding sites within the open reading frame (ORF) of the target mRNA. Animal microRNAs recognize partially complementary binding sites located in the 3′-untranslated region (3′-UTR). For most microRNA binding sites, the complementarity is limited to the seed sequence containing nucleotides #2–7 on the microRNA molecule. Note that in both plant and animal microRNAs the 5′-terminal nucleotide (#1) is not involved in target recognition.

In animals, microRNAs are processed from longer hairpin transcripts, known as pre-microRNA, by the RNase III-like enzymes Drosha and Dicer, whereas in plants only Dicer is responsible for microRNA processing [4,5]. One strand of the hairpin duplex is loaded into an Argonaute family protein (AGO) to form the core of microRNA-induced silencing complexes (miRISCs). miRISCs silence the expression of target genes through mRNA decay and translational repression. The target recognitions are achieved through base-pairing complementarity between the loaded microRNA and the target mRNA that contains a partially or fully complementary sequence [4,5]. Unlike plant microRNAs, that recognize fully complementary binding sites within the open reading frame (ORF), animal microRNAs recognize partially complementary binding sites generally located in the 3′-untranslated region (UTR) (Figure 1). For most microRNA binding sites, the complementarity is limited to the seed sequence found in the 5′-end of microRNA from nucleotide 2 to 7. The partial recognition between microRNA and its target is sufficient to trigger silencing.

The significance of microRNA in renal pathophysiology has been demonstrated in Dicer knockout animal models. During kidney development, the global knockout of Dicer in nephron progenitor cells results in a marked decrease in nephron number [6]. Conditional removal of Dicer from the ureteric lineage results in cystic kidney disease [7]. Podocyte-specific loss of Dicer function causes proteinuria, foot process effacement, and glomerulosclerosis [8–10]. The inducible deletion of another microRNA processing enzyme, Drosha, in mature podocytes from two- to three-month-old mice, results in a similar phenotype, demonstrating a post-developmental need for microRNA activity in podocytes [11]. Deletion of Dicer in renin-secreting juxtaglomerular cells results in a selective loss of these juxtaglomerular cells, suggesting a role in cell fate determination [12]. In the proximal tubule, microRNA appear to promote cellular injury because a selective loss of Dicer in animals after three weeks of age confers resistance to ischemia-reperfusion injury [13].

## 2. MicroRNA in Glomerular Diseases

Hypertrophy and expansion in the glomerular mesangium, along with podocyte dysfunction and accumulation of extracellular matrix proteins, are major features of diabetic nephropathy, glomerulonephritis, glomerulosclerosis, and many other types of glomerular pathologies. Studies of microRNAs and their targets in glomeruli will provide critical insights of the pathogenesis of glomerular diseases and reveal new therapeutic targets for pharmacological intervention.

### 2.1. MicroRNA in Glomerular Mesangial Cell

**Figure 2:**
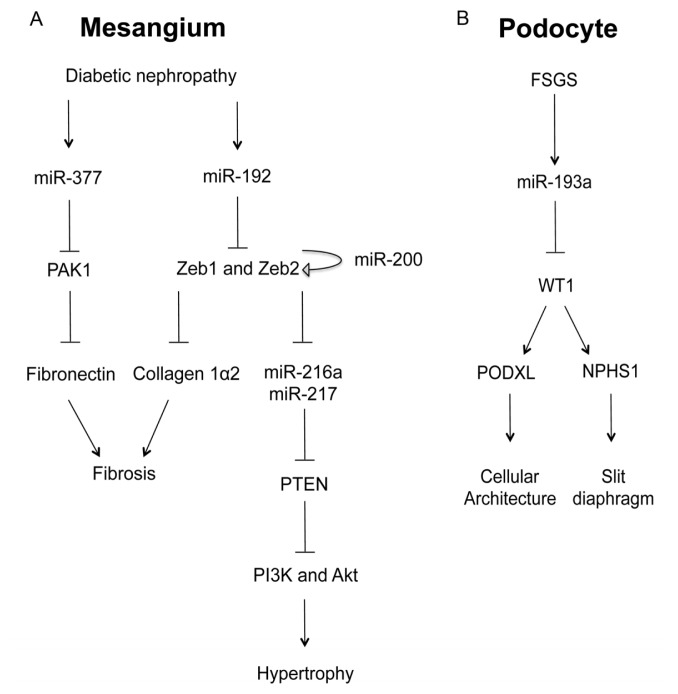
The role of microRNA in glomerular diseases. (**A**) In the mesangial cell, diabetic conditions increases the levels of miR-377 and miR-192, both of which promote fibrosis and hypertrophy through the signaling cascades involving PAK1 and Zeb1/2; (**B**) In the podocyte, focal segmental glomerulosclerosis (FSGS) induces the expression of miR-193a, which in turn inhibits WT1, a master regulator of podocyte homeostasis through podocalyxin (PODXL) and nephrin (NPHS1).

Among the microRNAs highly expressed in the kidney [14,15], several key microRNAs (miR-192, miR-200b, miR-200c, miR-216a, and miR-217) were upregulated in glomerular mesangium of diabetic mouse models (type I (streptozotocin (STZ)-induced) and type2 (db/db)) (*vide infra*). *In vitro*, TGF-β-induced miR-192 was shown to increase the gene expression of collagen 1α2 by reducing the expression of two E-box repressors (Zeb1 and Zeb2) that control collagen 1α2 gene activation [16]. *In vivo*, the miR-192 and Collagen 1α2 levels were substantially increased in the mesangial cells of STZ-induced diabetic mice, as well as of db/db diabetic mice, suggesting a role in glomerular basement membrane thickening (Figure 2A) [16]. miR-216a and miR-217 are downstream targets of miR-192 through Zeb1/2 mediated mechanisms [17]. In diabetic mesangium, the cellular levels of both microRNAs were increased, resulting in the silencing of their target—PTEN (phosphatase and tensin homolog). PTEN is a phosphatidylinositol-3,4,5-triphosphate (PIP3)-phosphatase that inhibits the phosphoinositide-3-kinase pathway (PI3K) and thus prevents Akt activation [18]. Akt activation by miR-216a and miR-217 led to glomerular mesangial cell expansion and hypertrophy, another hallmark of diabetic nephropathy (Figure 2A). MiR-377 was also upregulated in cultured human and mouse mesangial cells by glucose or TGF-β treatment or in type I diabetes [19]. Overexpression of miR-377 in mesangial cells *in vitro* increased fibronectin protein production, another component of the glomerular extracellular matrix. Mechanistically, miR-377 silenced the expression of serine/threonine protein kinase PAK1, also known as p21 protein (Cdc42/Rac)-activated kinase 1, and superoxide dismutase, both of which negatively controlled fibronectin protein production (Figure 2A) [19]. The miR-200 family members are separated into two clusters located in different genomic loci. Among them, miR-200b and c are regulated by the miR-192 targets—Zeb1/2 through E-boxes in the promoters of their host genes [20,21]. miR-200b and c also target the transcripts of Zeb1/2 to auto-regulate their own expression and amplify the signaling response leading to collagen expression and glomerular fibrosis (Figure 2A) [22].

IgA nephropathy is a leading cause of idiopathic glomerulonephritis and is characterized by mesangial deposition of IgA. The levels of glomerular microRNAs were deranged in patients with IgA nephropathy [23]. Among the analyzed microRNAs, the expression level of miR-200c was profoundly downregulated in these patients and negatively correlated with proteinuria, while the level of miR-192 was significantly upregulated and positively correlated with glomerulosclerosis [23].

### 2.2. MicroRNA in Glomerular Podocyte

Podocytes in the glomerular basement membrane are critical in the maintenance of structure and function of the glomerular filtration barrier. To study an overall role of miRNAs in podocyte biology, two independent lines of Dicer KO mice were generated for podocytes [9,10]. Mutant mice developed proteinuria by three weeks after birth and progressed rapidly to end-stage kidney disease. Multiple abnormalities were observed in glomeruli of mutant mice, including foot process effacement, irregular and split areas of the glomerular basement membrane, podocyte apoptosis and depletion, mesangial expansion, capillary dilation, and glomerulosclerosis [10]. Cytoskeletal dynamics was significantly altered in mutant animals, including early loss of synaptopodin and downregulation of the ERM protein family (ezrin-radixin-moesin) at three weeks [9]. Gene profiling revealed upregulation of 190 genes in glomeruli isolated from mutant mice at the onset of proteinuria. Target sequences for 16 microRNA were significantly enriched in the 3′-untranslated regions of 190 upregulated genes [10]. Bioinformatic approaches were used to validate six of the eight top-candidate microRNAs, which were miR-28, miR-34a, and four members of the miR-30 family (miR-30c-1, miR-30b, miR-30d, and miR-30c-2) [10]. The miR-30 family was shown to target four genes known to be functional in podocytes, including genes that mediate podocyte apoptosis (receptor for advanced glycation end product and immediate early response 3 protein) and cytoskeletal disruption (vimentin and heat shock protein 20).

Focal segmental glomerulosclerosis (FSGS) is a devastating glomerular diseases caused by podocyte dysfunction. Deranged expression of several podocyte specific genes (WT1, NPHS1, ACTN4, and TRPC6), accompanied by collapse of normal podocyte shape and podocyte foot process effacement, presents as major pathogenic origins for FSGS. The importance of microRNAs in FSGS has been demonstrated by Gebeshuber and colleagues in a recent study [24]. Through transgenic screening in mice, Gebeshuber *et al*. have identified miR-193a as a powerful inducer of FSGS. Mechanistically, miR-193a silences the Wilms’ tumor (WT1) gene, which encodes a transcriptional factor and acts as a master regulator for podocye homeostasis [24]. In normal podocytes, WT1 positively regulates the expression of several key genes crucial for podocyte architecture, e.g., podocalyxin (PODXL) and for slit diaphragm formation, e.g., nephrin (NPHS1). The level of miR-193a was consistently higher in isolated glomeruli from FSGS patients compared to normal kidneys, which provides an important mechanism for FSGS pathogenesis (Figure 2B) [24]. Deranged expression of selected microRNAs also causes podocyte abnormalities under diabetic conditions such as apoptosis and fibrosis [25,26]. In diabetic podocytes, the level of miR-195 is significantly elevated [25] while miR-29 is reduced [26]. MiR-195 targets the BCL2 gene and contributes to podocyte apoptosis via an increase in caspase-3 protein [25]. MiR-29, on the other hand, represses the expression of collagens I and IV, at both the mRNA and protein levels, by targeting the 3′-untranslated region of these genes [26].

## 3. MicroRNA in the Proximal Nephron

Renal ischemia-reperfusion injury (IRI) is a major cause of acute kidney injury (AKI), which is often associated with renal failure and high mortality rates [27]. The pathogenic role of microRNAs in AKI was interrogated by Wei *et al.* [13] using a proximal tubule specific Dicer KO model. Despite normal development, histology, and function of the kidney, these conditional KO mice are remarkably resistant to renal IRI, showing significantly better renal function, less tissue damage, lower tubular apoptosis rate, and higher survival rates. Microarray analyses in wildtype animals undergoing the same IRI procedure revealed changes in microRNA expression levels in the proximal tubule. Among the 173 microRNAs detected in renal cortex, miRNA-132, -362, -379, -668, and -687 showed continuous change during 12–48 h of reperfusion [13]. Another study by Godwin *et al.* demonstrated similar changes in microRNA expression during renal IRI in laboratory mice [28]. Consistent with the pathogenic hypothesis of microRNA in AKI, miR-34a was upregulated in mouse proximal tubular cells within a few hours of cisplatin-induced nephrotoxicity [29]. Inhibition of p53 with pifithrin-α abrogated the induction of miR-34a during cisplatin treatment. Ablation of miR-34a with antisense oligonucleotides led to increased apoptosis and reduced cell survival [29]. MiR-34a is therefore a novel downstream target of p53 in response to cisplatin induced DNA damage.

In contrast to its role in the glomerular mesangium, miR-192 appears to play a protective role against tubulointerstitial fibrosis in the proximal tubule of diabetic patients [30]. Krupa and colleagues performed a systematic study to profile microRNA expression levels in renal biopsies from patients with established diabetic nephropathy and identified 12 microRNAs showing significant differences from normal kidneys [30]. Among them, miR-192 showed the greatest change, the level of which was consistently lower in diabetic patients. In individual biopsies, miR-192 expression was inversely correlated with the progression of tubulointerstitial fibrosis and the loss of GFR. Mechanistically, overexpression of miR-192 in cultured proximal tubular cells suppressed the expression of Zeb1 and 2, opposing TGF-β-mediated epithelial-to-mesenchymal transition (EMT) [30].

## 4. MicroRNA in the Loop of Henle

**Figure 3:**
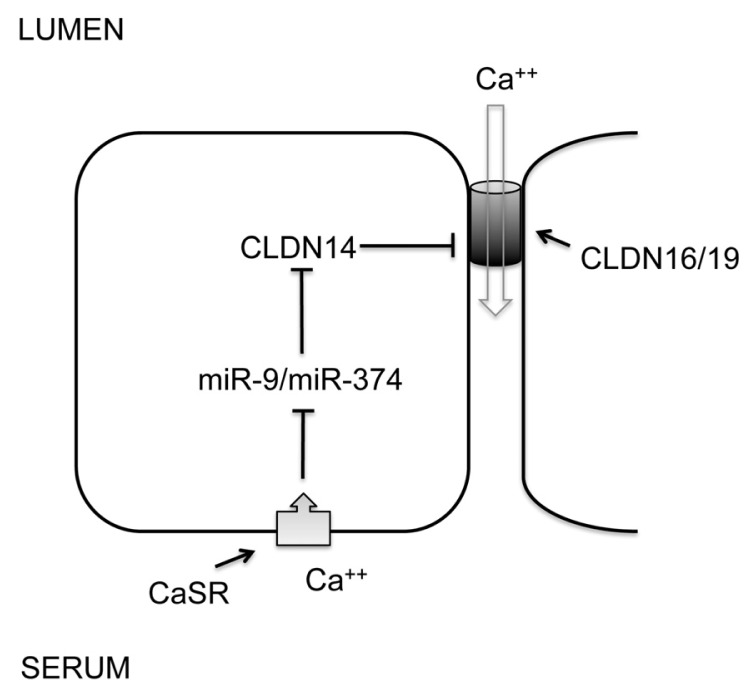
The role of microRNA in renal CaSR signaling. A feedback loop of CaSR signaling in the thick ascending limb of Henle’s loop (TALH) regulates urinary excretion of Ca^++^ through transcriptional regulation of miR-9 and miR-374. MicroRNAs in turn negatively regulate the expression level of claudin-14 through mRNA decay and translational repression. Claudin-14 directly binds to claudin-16 and inhibits its cation permeability to urinary Ca^++^.

The thick ascending limb of Henle’s loop (TALH) is responsible for extracellular Ca^++^ and Mg^++^ homeostasis. A signaling cascade made of CaSR and claudins senses extracellular Ca^++^/Mg^++^ differences and makes corresponding changes in renal excretion rates. CaSR—the Ca^++^ sensing receptor is a member of the G protein-coupled receptors (GPCRs). Mutations in the CaSR gene cause familial hypocalciuric hypercalcemia (FHH) and neonatal severe hyperparathyroidism (NSHPT), two inherited conditions characterized by altered calcium homeostasis [31]. Claudins are integral membrane proteins of the tight junction that is responsible for the paracellular transport of ions and solutes between apical and basolateral membranes. Mutations in the claudin genes, claudin-16 and claudin-19, cause familial hypomagnesemia with hypercalciuria and nephrocalcinosis (FHHNC) [32,33]. Synonymous sequence variants in the claudin-14 gene are associated with hypercalciuric kidney stones and reduced bone mineral density [34]. Several *in vitro* studies have revealed that that claudin-16 and -19 form a heteromeric cation channel in the TALH [35], whereas claudin-14 interacts with the claudin-16 and inhibits its cation permeability as a regulatory subunit [36]. Although the promoter activity and the mRNA level of claudin-14 are very high in the kidney, its protein level is surprisingly low. Gong *et al.* have identified two microRNA molecules, miR-9 and miR-374 from TALH cells, both of which recognize partially complementary binding sites located in 3′-UTRs of claudin-14 mRNA (Figure 3) [36]. Treatments with antisense oligonucleotide against miR-9 or miR-374 revealed that both microRNAs suppressed claudin-14 translation and induced its mRNA decay in a synergistic manner [36]. High Ca^++^ intake significantly downregulated the expression levels of miR-9 and miR-374 in TALH cells, which in turn causes a reciprocal increase in claudin-14 expression level. Deletion of CaSR from TALH cells abolished extracellular Ca^++^ induced changes in microRNA and claudin-14 [36]. The dietary regulation of microRNA suggests a physiological role for microRNA based signaling in the TALH of the kidney. The observed association between claudin-14 and hypercalciuric nephrolithiasis [34] can be explained by claudin-14 deregulation that escapes microRNA suppression, inhibits claudin-16/-19 channel permeabilities and phenocopies FHHNC to variable degrees. FHHNC patients [33,37] and animal models [38,39] with claudin-16 or claudin-19 mutations are known to have hypercalciuria, nephrocalcinosis, and nephrolithiasis. The regulation of microRNA by CaSR may occur on several layers: microRNA transcription, processing, or degradation [4]. Transcriptional regulation is undoubtedly the most specific way for individual microRNA. The promoters of both miR-9 (miR-9-3 locus) and miR-374 genes contain a canonical myc-binding site (E-box: CACGTG). The transcription of miR-9-3 is upregulated by myc in human breast cancer cells [40]; miR-421/-374 cluster is upregulated by myc in HeLa cells [41]. Although these studies did not prove a role for myc in CaSR signaling, both CaSR and claudin have been implicated in tumorigenesis and metastasis [42–44].

In addition to Ca^++^ metabolism, microRNAs also play a role in salt and fluid handling in the TALH. Mladinov *et al.* performed microarray assays in microdissected nephron segments and identified 12 microRNAs that were highly enriched in the TALH compared to the glomerulus and the proximal tubule [45]. Among them, miR-192 was found to target the Na^+^/K^+^-ATPase β1 subunit gene (Atp1b1). High salt diet increased the expression level of miR-192 in the TALH, which in turn suppressed Atp1b1 gene expression [45]. Knockdown of miR-192, *in vivo* with antisense oligonucleotide, upregulated the Atp1b1 protein level in the kidney, causing antidiuresis under high salt dietary condition. Contrasting with common microRNA target areas, miR-192 appeared to target Atp1b1 through the 5′-rather than 3′-untranslated region, although its binding sites were present within both regions [45].

## 5. MicroRNA in the Distal Nephron

The aldosterone-sensitive distal nephron (ASDN) encompasses the distal convoluted tubule (DCT), the connecting tubule, and the collecting duct. It is collectively responsible for the reabsorption of approximately 5% of filtered NaCl, and plays a vital role in the regulation of extracellular fluid volume (ECFV) and blood pressure [46]. The involvement of microRNA in hypertensive kidney disease was first studied by Sequeira-Lopez *et al.* in the juxtaglomerular cell [12]. The juxtaglomerular cell is found between the vascular pole of the renal corpuscle and the macula densa of the DCT. Its primary role in blood pressure control is the synthesis of renin. Renin is the key regulated step that initiates an enzymatic cascade that leads to angiotensin and aldosterone generation (collectively known as the rennin-angiotensin-aldosterone system). Conditional knockout of Dicer in juxtaglomerular cells resulted in a pronounced reduction in the number of juxtaglomerular cells accompanied by decreased expression of *Ren1* and *Ren2*, decreased plasma renin concentration, decreased blood pressure, and striking nephrovascular abnormalities, including striped corticomedullary fibrosis [12].

**Figure 4:**
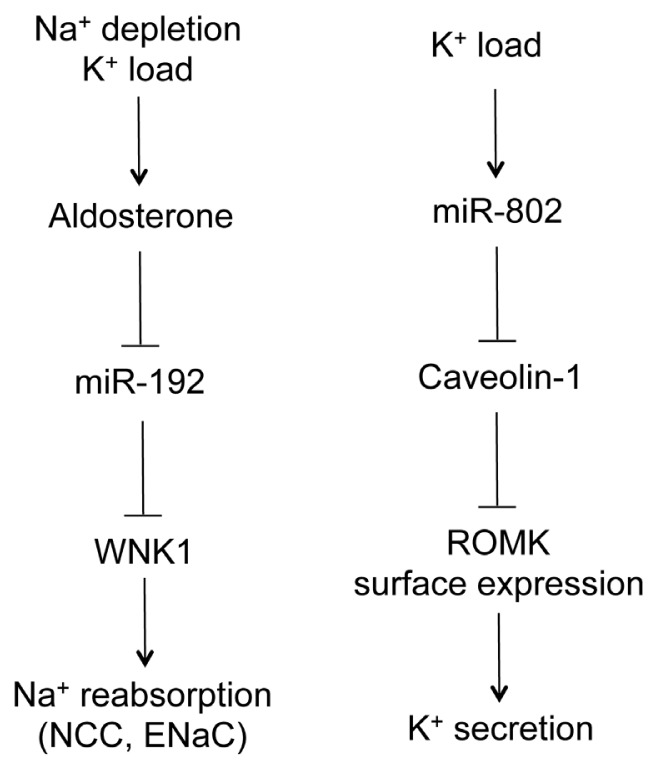
The role of microRNA in Na^+^ and K^+^ handling by the distal nephron. Na^+^ depletion or K^+^ load increases serum aldosterone level, which decreases miR-192 levels in the aldosterone-sensitive distal nephron (ASDN). MiR-192 suppresses WNK1 gene expression, which in turn regulates Na^+^ reabsorption through the NCC and ENaC channels. K^+^ load also increases miR-802 levels in the ASDN. MiR-802 suppresses caveolin-1 gene expression, which regulates renal outer medullary potassium (ROMK) channel surface expression through endocytosis. ROMK mediates K^+^ secretion to urine.

Two studies reported the role of microRNA in the regulation of ion transport by the ASDN. Elvira-Matelot *et al.* have shown that miR-192 expression is strongly reduced in the kidneys of mice treated with salt depletion, potassium load, or chronic aldosterone infusion, whereas its level is not modified by a high salt diet (Figure 4) [47]. The serine-threonine kinase WNK1 is the target of miR-192 when assayed *in vitro* and *ex vivo*. Its gene expression was reciprocally regulated by aldosterone, potassium and salt as to miR-192 in the kidney [47]. WNK1, belonging to the WNK (with no lysine-K) serine-threonine kinase subfamily, is essential for the coordinated regulation of Na^+^ and K^+^ transport in the kidney [48]. Mutations in WNK1 cause familial hyperkalemic hypertension (FHHt), a rare mendelian form of human hypertension [49].

The renal outer medullary potassium channel (ROMK) is an ATP-dependent potassium channel encoded by the gene Kir1.1 and responsible for K^+^ secretion along the ASDN. Its membrane localization is regulated by dietary K^+^ intake through endocytosis-mediated mechanisms. Lin *et al.* have revealed a physiological role for microRNA in ROMK endocytosis [50]. The ROMK channel is physically associated with caveolin-1, the principal protein component of caveolae. Expression of caveolin-1 varied inversely with the expression of ROMK in the plasma membrane, and caveolin-1 inhibited ROMK channel activity [50]. Caveolin-1 is the molecular target of miR-802 in the collecting duct. *In vitro*, expression of miR-802 suppressed the expression of caveolin-1; *in vivo*, the level of miR-802 was inversely correlated with that of caveolin-1 [50]. High K^+^ intake stimulated the transcription of miR-802, which in turn decreased the expression of caveolin-1 and increased the membrane localization of ROMK, leading to higher K^+^ excretion (Figure 4) [50].

MicroRNAs are also regulated by tonicity in the ASDN of the kidney. Exposure of the inner medullary collecting duct cells (mIMCD3) to a hypertonic solution induces a decrease in miR-200b and miR-717 expression level as early as two hours [51]. A common target for both microRNAs is the gene encoding the tonicity responsive element binding protein (TonEBP), also known as the osmotic response element binding protein (OREBP) [51]. TonEBP regulates many aspects of tonicity induced cellular responses, such as accumulation of inorganic osmolytes and expression of the HSP70 osmoprotective chaperone protein [52]. Depletion of microRNAs by knocking-down Dicer significantly increases TonEBP protein expression, while overexpression of miR-200b and miR-717 in mIMCD3 cells suppresses TonEBP expression on both the transcriptional and post-transcriptional levels [51]. *In vivo* in the mouse kidney, furosemide induced diuresis significantly upregulates miR-200b and miR-717, but downregulates TonEBP in the medulla [51]. The inverse correlation between the expression of microRNA and TonEBP suggests a signaling mechanism for renal cell adaptation to urinary osmolality.

## 6. MicroRNA as Therapeutic Candidates

Antisense oligonucleotides targeting specific microRNAs, termed “antagomirs”, were successfully used to silence endogenous microRNAs *in vivo* in experimental animals [53]. Antagomirs can be modified with methoxyethyl group (MOE) [53] or locked nucleic acid (LNA) [54] to enhance their *in vivo* stability. Once administered systemically, antagomirs are absorbed by major organs such as the liver, lungs, skin, spleen, lymph nodes, bone marrow, and kidney, excepting the brain [53–56]. The effect of a single intravenous dose of antagomir can last up to 21 d in liver and kidney [54,55]. MiR-21 regulates the ERK-MAPK signaling pathway to stimulate fibroblast survival and growth factor secretion [57]. Antagomirs against miR-21 was used to block fibrosis in cardiovascular diseases [57] and in pulmonary diseases [58]. The same strategy was also proven effective for treating renal fibrosis in chronic kidney diseases [57]. The liver-expressed miR-122 is essential for hepatitis C virus (HCV) RNA accumulation [59]. Antagomir treatments in nonhuman primates with chronic HCV infection generated long-lasting suppression of HCV viremia with no evidence of viral resistance or other adverse effects in the treated animals [60]. The most effective treatment of kidney diseases with antagomirs was demonstrated in the case of miR-192. Anti-miR-192 treatments ameliorated glomerular fibrosis in mouse models of diabetic nephropathy through a concomitant repression of collagen and fibronectin levels in the mesangial cells [61]. Another approach to interfere with the binding of a microRNA to its cognate target mRNA is the use of “microRNA sponge”. A “microRNA sponge” is a construct encoding an mRNA (e.g., the GFP mRNA) that contains a tandem repeat of microRNA binding sites in the 3′-UTR [62]. The sponges can be introduced *in vivo* to target organs via lentivirus or retrovirus mediated transgenesis.

The major limitation to microRNA therapeutics is the off-target effect, which occurs on two distinct levels. Most current miRNA modulators display a high degree of target specificity, as point mutations made into an antagomir can completely abolish its regulatory effect both *in vitro* and *in vivo* [63]. Nevertheless, the antagomir may still target a class of related microRNAs that differ only at one or two nucleotide loci. Additional ambiguity derives from the relatively modest inhibitory effects of individual microRNAs on mRNA targets. For example, a complete depletion of a microRNA often results in maximal increases (<1.5-fold) in the expression levels of its mRNA targets, suggesting that it is the cumulative impact of small changes in the expression of myriad targets—rather than pronounced changes in a single target—that mediates the biological actions of microRNAs. A more perplexing matter is the undesired effect of systemically administrated antagomirs in off-target organs or off-target cells in the target organ. Local administration of antagomirs represents a potential solution. For example, intracerebral injection of anti-miR-16 has been demonstrated to reduce miR-16 levels in the brain, which was, however, immune to any systemic manipulation [63]. Coupling antagomirs to tissue specific antibodies provides another means to manipulating microRNA levels locally. Upstream signals intervening with microRNA expression or maturation may be unique to each target organ, thus providing new layers of specificity in microRNA-based therapeutics. Despite these potential challenges, microRNA therapeutics have recently entered Phase I and Phase II clinical trials of Santaris Pharma’s antagomir anti-miR-122, *miravirsen*, for the treatment of HCV.

In some cases, a gain-of-microRNA function is beneficial to treating diseases. This can be achieved by use of a synthetic double-stranded precursor microRNA molecule, known as the “miRNA mimic”. In acute myocardial infarction, for example, miR-29 is significantly downregulated in fibroblast cells of the heart, resulting in cardiac fibrosis. Introducing miR-29 mimics profoundly reduced collagen expression and ameliorated fibrotic phenotypes in the heart [64].

MicroRNAs have also been found in blood and urine samples and have emerged as potential biomarkers for various diseases including kidney diseases [65]. Nevertheless, no association study is currently available to link the expression levels of these markers with disease progression and their power for predicting disease susceptibility is still debated.

## 7. Conclusions

MicroRNA has emerged to be a critical regulator underlying a diverse range of renal pathophysiologies. Owing to its short seed sequence, a cognate microRNA regulates multiple gene targets, making it a powerful signaling molecule to coordinate various cellular functions. Manipulation of tissue microRNA level as a novel therapeutic approach has been proven effective in several renal disease models. Systematic identification of downstream microRNA target genes will improve our knowledge in renal pathology and supply additional candidates for therapeutic intervention. What remain largely unknown are the mechanisms that endogenously control microRNA metabolism and the clinical evidence that derangement of such mechanisms causes human diseases.
